# Acute polyarthritis in a young patient caused by meningococcal and parvovirus B19 infections: a case report and review of the literature

**DOI:** 10.1186/s13256-016-1156-4

**Published:** 2016-12-20

**Authors:** Virginie Lavoipierre, Anna Dellyes, Camille Aubry, Christine Zandotti, Pierre Lafforgue, Philippe Parola, Jean-Christophe Lagier

**Affiliations:** 1 Service de Maladies Infectieuses Aigues, Pôle Maladies Infectieuses, Assistance Publique Hôpitaux de Marseille, CHU Timone, Institut Hospitalo-Universitaire Méditerranée Infection, 13005 Marseille, France; 2 Service de Rhumatologie, Assistance Publique Hôpitaux de Marseille, CHU Sainte Marguerite, 13009 Marseille, France; 3 Pôle Maladies Infectieuses, Fédération de Microbiologie, Assistance Publique Hôpitaux de Marseille, CHU Timone, Institut Hospitalo-Universitaire Méditerranée Infection, 13015 Marseille, France; 4 Aix Marseille Université, URMITE, UM63, CNRS 7278, IRD 198, INSERM 1095, Faculté de Médecine, Institut Hospitalo-Universitaire Méditerranée-Infection, Marseille, France

**Keywords:** Meningococcemia, Polyarthritis, Parvovirus B19, Case report

## Abstract

**Background:**

Meningococcal infection is a multifaceted disease including acute polyarthritis. This presentation should be known by clinicians in order to prevent delay in treatment. We report what we believe to be the first case of an association of parvovirus B19 and meningococcal polyarthritis in a young adult.

**Case presentation:**

A 19-year-old Caucasian woman presented to our hospital with fever, intense leg pain, and a transient rash. A physical examination showed asymmetric polyarthritis and no neurological abnormalities. A parvovirus B19 polymerase chain reaction performed using a blood sample and knee fluid aspirate came back positive, but serology was negative for immunoglobulin M and positive for immunoglobulin G. A blood culture was positive for serotype C meningococcus; a polymerase chain reaction performed for *Neisseria meningitidis* was positive in joint fluid but negative in blood samples (performed after antibiotic treatment had begun). Our patient was treated with ceftriaxone for 15 days, associated with analgesic therapy. Hydroxychloroquine treatment was introduced 5 months after the onset of polyarthritis because of persisting inflammatory arthralgia.

**Conclusions:**

To the best of our knowledge, this is the first case report of polyarthritis caused by concomitant meningococcal and parvovirus B19 infections. This unusual presentation of meningococcal disease may have resulted from the persistent parvovirus B19 infection. Our experience with this case illustrates the need for a systematic approach to the diagnosis of febrile acute polyarthritis. Only long-term follow-up will reveal if this infectious polyarthritis will evolve towards an autoimmune rheumatism.

## Background

*Neisseria meningitidis* is a Gram-negative coccus that is restricted to humans. Asymptomatic nasopharyngeal carriage is frequent and infections are most frequent among infants and children under the age of 5 years, adolescents and young adults, and adults over the age of 65. Meningococcemia (19–40%) and meningitis (41–57.7%) [1] are the most common events that can be life-threatening and have severe long-term consequences. Nevertheless, meningococcal disease has a very broad spectrum of clinical manifestations that are less known to physicians and that can lead to misdiagnosis and delays in treatment. Parvovirus B19, which causes infections worldwide, has a tropism for erythroid precursor cells. Infection usually occurs in young children and tests for anti-parvovirus B19 immunoglobulin G (IgG) are positive in 50–60% of older children (over the age of 5 years), young adults, and women of child-bearing age [2]. The disease is mainly transmitted through the respiratory tract, although vertical and blood-borne transmission has also been described. Sub-clinical infections are common but parvovirus B19 infections can also cause erythema infectiosum, arthropathies, and hydrops.

In this study, we present a case of acute polyarthritis in a young adult woman with meningococcemia and a likely persistent infection by parvovirus B19. According to our literature search, it is the 21st case to be reported of polyarticular arthritis caused by *N. meningitidis* and the first report of concomitant meningococcal and parvovirus B19 infections.

## Case presentation

A 19-year-old Caucasian woman was admitted to our hospital for intense leg pain with fever and a transient rash on her lower body. With the exception of the birth control pill, she was not taking any regular medication and had no relevant past medical history, but her family history revealed several cousins with inflammatory rheumatism. She had been to Spain 3 weeks before and had stayed in an amusement park. There, she had no contact with animals with the exception of mosquito bites and her close relatives were not ill. She had last had sexual intercourse 3 years before, which was protected. Her vaccination status was unknown. Our patient had been suffering from headache, myalgia, fever, and acute severe abdominal pain for a few days. She first presented to our emergency department where an abdominal computed tomography scan was performed (no abnormalities) and blood tests were conducted (including blood cultures). She was prescribed acetaminophen and was discharged. When her symptoms persisted the next day, she returned to our emergency department. Upon admission, her temperature was 38.3 °C. She presented with tachycardia and a physical examination showed asymmetric polyarthritis affecting her major joints (knees, right ankle, and shoulder), non-specific lymphadenopathy, and a non-purpuric macular eruption of her legs and lower abdomen that resolved rapidly. Results of a neurological examination were normal and she showed no meningeal signs. Blood tests revealed a high white blood count (22 g/L with 20 g/L neutrophils), a procalcitonin concentration of 36.97 μg/L (normal range < 0.10 μg/L), and a C-reactive protein concentration of 291 mg/L (normal range 0–5 mg/L). Results from renal and liver function tests were normal. A cerebrospinal fluid analysis revealed no signs of meningitis (white blood cell (WBC) count of 4/mm^3^; glycorrhachia and proteinorachia were normal). Empiric antibiotic treatment with ceftriaxone was started as well as symptomatic treatment involving opioid titration.

Our patient was admitted to our infectious diseases department and then transferred to our rheumatology department. Supplementary analyses were performed and polymerase chain reaction (PCR) results were negative for hepatitis A, B, C viruses; human immunodeficiency virus; Lyme disease; syphilis; *Mycoplasma pneumoniae*; cytomegalovirus; Epstein–Barr virus; herpes simplex virus; chicken pox virus serologies; and chikungunya virus. PCR for parvovirus B19 was positive in the blood twice but serology showed negative results for immunoglobulin M (IgM) and positive for IgG (test controlled after 1 week). A knee fluid aspiration was performed, revealing sterile inflammatory joint liquid (WBC 18,000/mm^3^). PCR for parvovirus B19 was positive in the joint fluid. Autoimmune blood tests showed increased or normal complement component serum levels (C3 = 1.72 g/L, limits 0.81–1.57; CH50 > 143%, limits 70–130%; C4 = 0.32 g/L, limits 0.13–0.39 g/L), slightly positive cardiolipin antibodies (44 units/mL), and positive antinuclear antibodies (1:640). No joint damage was observed on X-ray. Finally, blood culture (the first sample drawn upon arrival) was positive for serotype C meningococcus. PCR for *N. meningitidis* was then positive in her joint fluid, but negative in a blood sample (performed after antibiotic treatment had begun).

Our patient evolved toward apyrexia along with a decrease in systemic inflammation markers but persistent, painful arthritis. In addition, her disease evolution was complicated by deep vein thrombosis in her left leg. We continued antibiotic treatment with ceftriaxone for a total duration of 15 days. Anticoagulation therapy was also started for the phlebitis. Analgesia was difficult to obtain and our patient needed a combination of acetaminophen, opioid, and nonsteroidal anti-inflammatory drugs. After 3 weeks, nonsteroidal anti-inflammatory drugs were replaced with corticotherapy at 15 mg per day for 1 month before being tapered. One month after admission, opioids were stopped. Out patient was pain free, had no arthritis, and had gone back to her daily routine. Unfortunately, corticosteroids could not be discontinued because the pain returned when our patient took <7 mg of prednisone a day. Hydroxychloroquine treatment was introduced 5 months after the onset of polyarthritis. Treatments were well tolerated.

## Discussion

To the best of our knowledge, this is the first report of a case of polyarthritis probably caused by concomitant meningococcal and parvovirus B19 infections. Indeed, we report a rare manifestation of meningococcal infection, perhaps resulting from a persistent parvovirus B19 infection. We are confident in our results because meningococcal disease was documented by both blood culture and PCR performed on joint fluid and parvovirus B19 was documented by PCR performed on both blood and joint fluid. In addition, we used stringent protocols for PCR, including negative and positive controls.

Arthritis is an uncommon manifestation of meningococcal infection. Cabellos *et al.* [3] assessed the clinical characteristics of arthritis related to invasive meningococcal disease. They described 39 cases of meningococcal arthritis out of 522 cases of invasive meningococcal disease (7.5%). Several results were statistically significant: meningococcal arthritis was less frequently associated with meningitis than with other manifestations of meningococcal disease (79% versus 91%, odds ratio (OR) 0.41, 95% confidence interval (CI) 0.17–0.94, p = 0.003) and, unlike in our patient’s case, septic arthritis was more often monoarticular (11 out of 15 versus 8 out of 23, OR 5.15, 95% CI 1.2–2.1, p = 0.02) and occurred in younger people (median age 22 years versus 39 years, OR 1.04, 95% CI 1.00–1.08, p = 0.02). Moreover, skin lesions were observed in 95% of patients and arthritis more frequently involved large joints. Finally, in their study, blood cultures were positive in approximately half of the cases and serogroup B meningococcus was predominant. Different kinds of arthritis associated with meningococcal disease have been reported. For example, Schaad *et al.* distinguished arthritis occurring as a complication of acute meningococcal disease, primary meningococcal arthritis (as in our case report), or arthritis in chronic meningococcemia [4]. We found 20 case reports of primary meningococcal polyarticular arthritis in adults and children from 1977 to 2015 (Table 1). Diagnoses were made by positive blood culture or positive joint fluid culture. Among them, one case that only included a positive pharyngeal swab test was considered to be uncertain. In our literature review, *N. meningitidis* was found in both blood and joint fluid, as in our patient, in only two patients. The predominance of one particular serogroup in meningococcal arthritis is still unclear. Contradictory results have previously been reported, such as the association of meningococcal arthritis and serogroup W135, which was statistically significant for Vienne *et al.* [5], whereas serogroup B was predominant for Cabellos *et al.* [3] and serogroup C for Gottfredsson *et al.* [6].

**Table 1:** Case reports of primary meningococcal polyarthritis in the literature from 1977 to 2015

Patient no.	Authors	Year	Age	Sex	Rash	Respiratory symptoms	Joint fluid	Blood culture (serogroup)
1	Sarinho *et al.* [13]	2015	53 years	F	+	+	Not performed	+ (C)
2	Bouayed *et al.* [14]	2012	7 months	F	+	–	Not performed	+ (B)
3	Verma *et al.* [15]	2011	19 years	M	+	+	Gram-negative cocci, PCR +	–
4	Stryhn and Haller [16]	2010	4 years	M	UNK	–	+	–
5	Germino *et al.* [17]	2009	7 years	M	–	+	UNK	+ (Y)
6	Bookstaver and Rudisill [18]	2009	38 years	M	UNK	UNK	+	+ (X)
7	Harwood *et al.* [19]	2008	29 years	F	+	–	Gram-negative cocci	+
8	McCulloch *et al.* [20]	2008	19 years	M	UNK	+	Inflammatory	UNK
9	Davis *et al.* [11]	2007	19 years	F	+	+	Inflammatory	+ (B)
10	Bongers *et al.* [21]	1998	35 years	F	+	–	Purulent fluid	–
11	Dillon *et al.* [22]	1997	12 years	M	+	–	–	+ (C)
12	Omar *et al.* [23]	1994	52 years	M	UNK	–	UNK	UNK
13	Travis *et al.* [24]	1989	15 years	M	+	+	+	–
14	Salmeron *et al.* [25]	1986	40 years	M	+	+	+	–
15	Kidd *et al.* [26]	1985	17 years	M	+	+	-	–
16	Kidd *et al.* [26]	1985	17 years	F	+	–	Not performed	+ (B)
17	Kidd *et al.* [26]	1985	14 years	M	+	+	Not performed	+ (C)
18	Pinals [27]	1977	UNK	UNK	–	UNK	–	+
19	Pinals [27]	1977	UNK	UNK	–	UNK	–	+
20	Pinals [27]	1977	UNK	UNK	–	UNK	–	+

*F* female, *M* male, *UNK* unknown, *PCR* polymerase chain reaction

In adults, peripheral polyarthritis is the most common manifestation of parvovirus B19 infection (60% of women and 30% of men), while skin lesions are not as frequent or typical as in children [7, 8]. Parvovirus B19 acute polyarthritis can mimic rheumatoid arthritis but one important discriminating feature is the absence of erosive lesions in parvovirus infections [7]. In most patients, polyarthritis resolves after 2–3 weeks but chronic arthritis has been described with parvovirus B19. Metacarpophalangeal joints (75%), knees (65%), wrists (55%), and ankles (40%) are the most frequently involved joints [7]. The mechanism is thought to be immunologically mediated and significant morning stiffness is often present [8]. In our case, several different samples were positive for parvovirus B19. However, although PCR for parvovirus B19 was positive, tests for IgM were negative and her serology was more consistent with a prior infection. The persistence of parvovirus B19 deoxyribonucleic acid (DNA) in joint fluid following a prior infection could explain these findings. Several studies detected the persistence of parvovirus B19 DNA in asymptomatic patients. Corcioli *et al.* [9] observed that 51% of the patients included in their cohort had persistent parvovirus B19 DNA, more often in solid tissues (particularly synovium) than in bone marrow. Furthermore, one case report described ascorbic acid efficiency against chronic arthralgia caused by persistent parvovirus B19 viremia [10]. This patient had positive PCR in blood samples and positive results for IgG, 11 and 13 months respectively after the onset of symptoms. In the case of our patient, we suspect that parvovirus B19 asymptomatic chronic infection may have increased the risk of joint localization of *N. meningitidis* following the bacteremia, by affecting her immune system and the inflammatory reaction in her joints. Other cases of co-infection with *Neisseria meningitidis* can be found in the literature, such as Epstein–Barr virus co-infection [11, 12].

## Conclusions

The aim of our report was to present a case of primary meningococcal arthritis, which is a rare manifestation of meningococcal disease often leading to wrong or delayed diagnosis, and to discuss its unusual association with parvovirus B19, another cause of acute polyarthritis. Only long-term follow-up will reveal whether these infections will evolve toward autoimmune rheumatism as suggested by our patient’s family history and the positivity of autoantibodies.

## Abbreviations

Ig: Immunoglobulin
OR: Odds ratio
CI: Confidence interval
PCR: Polymerase chain reaction
WBC: White blood cells
